# Decision self-efficacy and its determinants among Chinese adults with pneumoconiosis: a cross-sectional study

**DOI:** 10.3389/fpubh.2026.1843585

**Published:** 2026-06-30

**Authors:** Chenxi Zhong, Xiangwen Gong, Kaiwang Cui, Ting Liu, Hongwang Liu, Yanqing Lv

**Affiliations:** 1 Ganzhou Key Laboratory of Respiratory Diseases, Ganzhou Institute of Respiratory Diseases, Ganzhou Fifth People's Hospital Aliated to Gannan Medical University, Ganzhou, Jiangxi, China; 2 Department of Respiratory and Critical Care Medicine, Ganzhou Key Laboratory of Respiratory Diseases, Ganzhou Institute of Respiratory Diseases, Ganzhou Fifth People's Hospital Affiliated to Gannan Medical University, Jiangxi, China; 3 Hospital Infection Control Department, Ganzhou Key Laboratory of Respiratory Diseases, Ganzhou Institute of Respiratory Diseases, Ganzhou Fifth People's Hospital Affiliated to Gannan Medical University, Ganzhou, Jiangxi, China

**Keywords:** decision-making self-efficacy, influencing factor, occupational disease, pneumoconiosis, survey studys

## Abstract

**Background:**

Decision self-efficacy is a key component of effective self-management in chronic diseases, yet little is known about its status and determinants among patients with pneumoconiosis. This study aimed to assess decision-making self-efficacy and identify its socio-demographic and clinical predictors in Chinese adults with pneumoconiosis.

**Methods:**

A cross-sectional survey was conducted among 268 patients with pneumoconiosis recruited from a tertiary hospital in Ganzhou, China. Data were collected using a general demographic questionnaire and the Chinese version of the Decision-Making Self-Efficacy Scale. Independent-samples *t*-tests, one-way analysis of variance, and multiple linear regression analyses were performed to assess the levels and determinants of decision-making self-efficacy, including interactions between key socio-economic factors.

**Results:**

The mean decision-making self-efficacy score among patients with pneumoconiosis was 56.75 ± 17.54, indicating a moderate-to-low level. Greater decision-making self-efficacy was significantly associated with higher educational level and higher monthly income. In contrast, advanced disease stage, being widowed or divorced, home oxygen therapy, and anxiety were linked to lower self-efficacy. A significant interaction between education and income indicated that the beneficial effect of education was amplified among higher-income patients (*P* for interaction = 0.033).

**Conclusion:**

Decision-making self-efficacy among patients with pneumoconiosis is suboptimal and influenced by socio-economic, clinical, and psychosocial factors. Individualized decision-support strategies should be developed to enhance patients' decision-making self-efficacy and promote active participation in treatment decision-making, particularly for patients with lower education or income, advanced disease, or limited social support.

## 1 Introduction

Pneumoconiosis is an incurable but preventable interstitial lung disease caused by the occupational inhalation and retention of mineral dust particles, such as crystalline silica, coal dust, and asbestos fibers (1). The disease is characterized by a long latency period, a chronic and progressive course, and irreversible pulmonary damage (2). Affected individuals often require long-term comprehensive treatment and follow-up management, which substantially impairs health-related quality of life and social functioning. As the world's largest producer and consumer of coal, China also has the largest population of coal miners and the highest number of pneumoconiosis cases, resulting in a disproportionately high global disease burden (3). According to the Global Burden of Disease Study 2019, China accounts for more than two-thirds of global incident cases of pneumoconiosis and related disability-adjusted life years (DALYs) (4). Furthermore, the 2022 national occupational disease report indicated that pneumoconiosis cases constituted nearly 90% of all reported occupational diseases in China (5). Collectively, these data underscore the persistently severe public health challenge posed by pneumoconiosis in China.

Patients with pneumoconiosis typically live with prolonged disease progression and limited therapeutic reversibility, facing complex and evolving treatment trajectories that involve multiple medical and health-related decision points. These decisions often require patients to weigh potential benefits and risks under conditions of uncertainty. With the ongoing advancement of the “Healthy China” initiative, healthcare delivery in China has increasingly shifted toward a patient-centered paradigm, emphasizing shared decision-making between healthcare professionals and patients and encouraging active patient involvement in disease treatment and management (6). In this context, patients' capacity and confidence to participate in medical decision-making have become critical determinants of decision quality. Decision-making self-efficacy refers to an individual's confidence in their ability to understand relevant information, evaluate available options, and make informed healthcare decisions. It has been shown to predict patients' willingness to actively engage in the decision-making process and is recognized as a key intervention target for enhancing patient participation in clinical care (6). Previous studies have demonstrated the relevance of decision-making self-efficacy across a range of healthcare contexts, including renal replacement therapy (7), vaccination decision-making (8), and prostate cancer screening (9). Higher levels of decision-making self-efficacy are associated with more active patient–provider communication, more informed choices, and reduced decisional conflict and decisional regret (9).

Despite the substantial disease burden and complex decision-making demands faced by patients with pneumoconiosis, empirical evidence regarding their decision-making self-efficacy remains scarce. The research questions of this study are: (1) What is the level of decision-making self-efficacy among Chinese adults with pneumoconiosis? (2) Which socio-demographic, clinical, and psychosocial factors are associated with decision-making self-efficacy in this population? Correspondingly, the study hypothesizes that decision-making self-efficacy is at a moderate-to-low level, and that these factors could influence self-efficacy, without presuming the direction or magnitude of these associations.

To our knowledge, this study is the first to assess the current status of decision-making self-efficacy among patients with pneumoconiosis in the Gannan region of China and to identify its associated influencing factors. A better understanding of decision-making self-efficacy in this population may provide an important empirical basis for developing targeted and standardized decision-support interventions, facilitating shared decision-making processes, and ultimately improving the quality of healthcare decisions for patients with pneumoconiosis.

## 2 Methods

### 2.1 Study design and participants

This cross-sectional study enrolled hospitalized patients with pneumoconiosis at Ganzhou Fifth People's Hospital from January 2025 to April 2026. Pneumoconiosis was diagnosed according to the Chinese national diagnostic criteria (GBZ 70–2015). Eligible participants were aged 18–80 years and provided written informed consent. Patients were excluded if they had severe psychiatric disorders, serious comorbid conditions that could impair participation, or other clinical circumstances considered unsuitable by investigators.

This study was approved by the Ethics Committee of Ganzhou Fifth People's Hospital (GZWY-EC-科审-2024023).

The sample size was determined based on requirements for multivariable regression. Considering approximately 17 candidate independent variables and a recommended minimum of 10–15 participants per variable, the minimum required sample size ranged from 170 to 255. After inflating the estimate by 20% to account for potential exclusions and missing data, the target sample size was 204–306. A total of 300 patients were initially recruited. Of these, 32 were excluded because of severe psychiatric disorders (*n* = 4), serious comorbid conditions (*n* = 11), or incomplete data (*n* = 17), leaving 268 participants for the final analysis.

### 2.2 Measurement of decision self-efficacy

Decision self-efficacy was assessed using the Decision Self-Efficacy Scale developed by O'Connor (23), which consists of 11 items assessing patients' confidence in making informed medical decisions (Supplementary Table 1). Each item is rated on a 5-point Likert scale ranging from 0 (strongly disagree) to 4 (strongly agree). Total scores were standardized to a 0–100 scale, with higher scores indicating greater decision self-efficacy. The scale has shown acceptable validity and reliability in prior studies (10).

### 2.3 Covariates

Demographic and clinical variables were collected using structured questionnaires and medical records. These included age, gender, residence, marital status, education level, smoking history, healthcare payment type, monthly income, disease duration, pneumoconiosis stage, comorbidity status, rehabilitation exercise participation, home oxygen therapy, anxiety, and depression. Residence was categorized as urban, town, or rural. Education level was classified as primary school or below, junior high school, and high school or above. Healthcare payment type included employee medical insurance, resident medical insurance, and out-of-pocket payment. Monthly income was categorized as < 3,000, 3,000–5,000, 5,000–10,000, and >10,000 yuan. Anxiety and depression were assessed using the Self-Rating Anxiety Scale (SAS) and the Self-Rating Depression Scale (SDS), respectively. Standard cutoff scores (SAS ≥50; SDS ≥53) were used to define significant anxiety and depression.

### 2.4 Statistical analysis

Continuous variables are presented as mean ± standard deviation (SD), and categorical variables as frequencies and percentages. Score distributions were assessed visually using histograms and evaluated for normality using the Shapiro-Francia test.

Differences in decision self-efficacy scores across categorical variables were assessed using independent-samples *t*-tests or one-way analysis of variance (ANOVA), as appropriate. Multivariable linear regression was performed to identify factors independently associated with decision self-efficacy. Variables with *P* < 0.05 in univariate analysis were included in the multivariable model. Regression coefficients (β) and 95% confidence intervals (CIs) were reported.

The interaction between education level and monthly income was examined by including cross-product terms in the regression model. The statistical significance of interactions was evaluated using the Wald test. Adjusted predictive margins and 95% CIs were estimated using the margins command and visualized using marginsplot.

All analyses were conducted using Stata/SE 15.0 (StataCorp LLC, College Station, TX, USA). A two-sided *P* < 0.05 was considered statistically significant.

## 3 Results

### 3.1 Participant characteristics

A total of 268 patients were included in the analysis. Baseline characteristics are shown in Table 1. Most participants were more than 60 years (85.07%), male (95.15%), and rural residents (44.78%). The majority were married (92.91%) and covered by resident medical insurance (86.57%). Regarding disease characteristics, 39.18% were classified as stage II pneumoconiosis, and 41.04% had comorbid conditions. Anxiety and depression were present in 61.94 and 59.33% of participants, respectively.

**Table 1:** Baseline characteristics in Chinese adults with pneumoconiosis (*n* = 268).

Characteristics	Options	*n* (%)
Age	< 60 years old	40 (14.93)
	≥60 years old	228 (85.07)
Gender	Male	255 (95.15)
	Female	13 (4.85)
Residence	Urban	53 (19.78)
	Town	95 (35.45)
	Rural	120 (44.78)
Marital status	Married	249 (92.91)
	Other Widowed/Divorced	19 (7.09)
Education	Primary school and below	14 (5.22)
	Junior high school	97 (36.19)
	High school or above	157 (58.58)
Smoking history	No	105 (39.18)
	Yes	163 (60.82)
Healthcare payment	Employee Medical Insurance	22 (8.21)
	Out-of-pocket	14 (5.22)
	Resident Medical Insurance	232 (86.57)
Monthly income	< 3000 yuan	40 (14.93)
	3000–5000 yuan	163 (60.82)
	5000–10000 yuan	52 (19.40)
	>10000 yuan	13 (4.85)
Disease course	0–5 years	105 (39.18)
	6–10 years	109 (40.67)
	>10 years	54 (20.15)
Stage	Stage I	86 (32.09)
	Stage II	105 (39.18)
	Stage III	77 (28.73)
Comorbidity	No	158 (58.96)
	Yes	110 (41.04)
Rehabilitation exercise	Never	121 (45.15)
	Occasionally	94 (35.07)
	Regularly	53 (19.78)
Home oxygen therapy	No	244 (91.04)
	Yes	24 (8.96)
Anxiety (SAS)	No	102 (38.06)
	Yes	166 (61.94)
Depression (SDS)	No	109 (40.67)
	Yes	159 (59.33)

### 3.2 Distribution of decision self-efficacy scores

**Figure 1:**
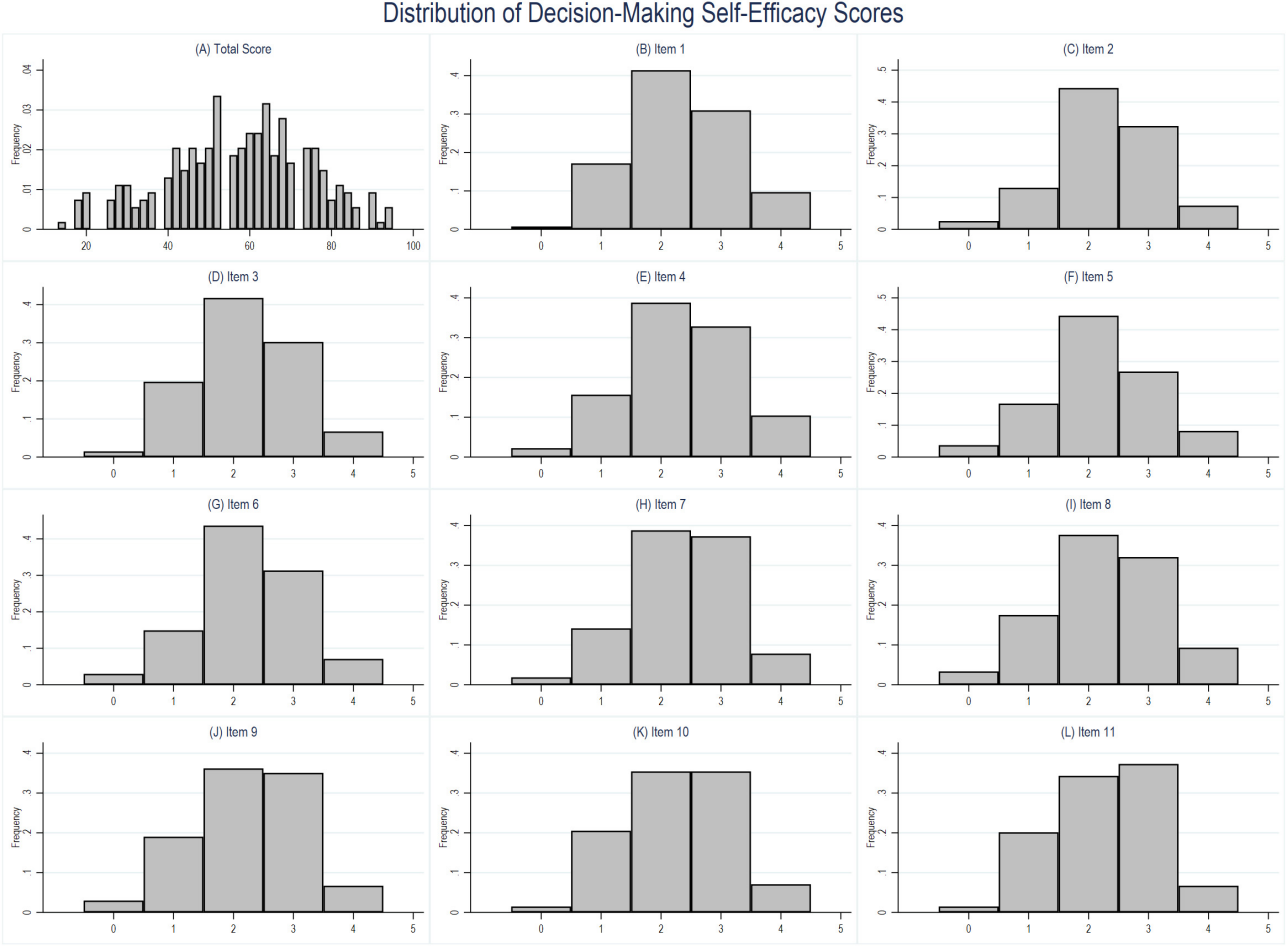
Distribution of decision self-efficacy scores. **(A)** Total score; **(B)** Item 1 – Get accurate information about my possible medical options; **(C)** Item 2 – Get information about the benefits of each option; **(D)** Item 3 – Get information about the risks and side effects of each option; **(E)** Item 4 – Be fully informed and able to make a choice; **(F)** Item 5 – Ask questions without feeling hesitant; **(G)** Item 6 – Express my concerns about each treatment option; **(H)** Item 7 – Actively seek support; **(I)** Item 8 – Understand which treatment option is best for me; **(J)** Item 9 – Handle unnecessary pressure from others when making a choice; **(K)** Item 10 – Let the healthcare team know what is best for me; **(L)** Item 11 – Delay the decision if I feel I need more time.

The Shapiro-Francia test indicated that both total scores and individual item scores followed approximately normal distributions (all *P* > 0.05). The distribution of decision self-efficacy scores is shown in Figure 1 and Supplementary Table 2. The mean total score was 56.75 ± 17.54. Among individual items, the highest item scores were observed for actively seeking support (2.35 ± 0.88) and being fully informed (2.34 ± 0.94), whereas the lowest item score was observed for asking questions without hesitation (2.19 ± 0.94) and understanding risks and side effects (2.21 ± 0.89).

### 3.3 Univariate analysis

Univariate analyses are presented in Table 2. Higher decision self-efficacy scores were observed among male participants, urban residents, those with higher education and higher monthly income, shorter disease duration, earlier pneumoconiosis stage, being married, absence of comorbidities, regular rehabilitation exercise, and absence of anxiety or depression (all *P* < 0.05). In contrast, age and smoking history were not significantly associated with decision self-efficacy (both *P* > 0.05).

**Table 2:** Univariate analysis of factors associated with decision self-efficacy in Chinese adults with pneumoconiosis.

Characteristics	Options	Total score (Mean ±SD)	t / F	*P*
Age	< 60 years old	56.63 ± 17.82	0.263	0.792
	≥60 years old	57.42 ± 15.98		
Gender	Female	68.92 ± 19.08	2.593	0.010
	Male	56.13 ± 17.27		
Residence	Urban	66.15 ± 14.83	15.68	< 0.001
	Town	58.52 ± 16.95		
	Rural	51.19 ± 17.14		
Marital status	Married	58.53 ± 16.11	6.474	< 0.001
	Other Widowed/Divorced	33.37 ± 19.09		
Education	Primary school and below	48.54 ± 14.90	65.02	< 0.001
	Junior high school	66.93 ± 13.97		
	High school or above	78.14 ± 11.41		
Smoking history	No	57.22 ± 17.95	0.357	0.721
	Yes	56.44 ± 17.32		
Healthcare payment	Employee Medical Insurance	69.00 ± 16.57	7.70	< 0.001
	Out-of-pocket	63.50 ± 20.18		
	Resident Medical Insurance	55.18 ± 16.97		
Monthly income (CNY)	< 3000 yuan	47.10 ± 17.62	15.99	< 0.001
	3000–5000 yuan	54.88 ± 15.35		
	5000–10000 yuan	65.40 ± 17.33		
	>10000 yuan	75.23 ± 17.94		
Disease course	0–5 years	60.12 ± 17.15	6.05	0.003
	6–10 years	56.79 ± 17.26		
	>10 years	50.09 ± 17.22		
Stage	Stage I	68.63 ± 12.98	62.16	< 0.001
	Stage II	56.92 ± 15.12		
	Stage III	43.23 ± 15.30		
Comorbidity	No	60.29 ± 17.18	4.083	< 0.001
	Yes	51.65 ± 16.86		
Rehabilitation exercise	Never	50.14 ± 16.34	18.40	< 0.001
	Occasionally	60.98 ± 18.67		
	Regularly	64.32 ± 12.13
Home oxygen therapy	No	58.22 ± 16.94	4.509	< 0.001
	Yes	41.89 ± 16.92		
Anxiety (SAS)	No	69.59 ± 11.98	11.458	< 0.001
	Yes	48.86 ± 15.67		
Depression (SDS)	No	68.87 ± 11.93	11.412	< 0.001
	Yes	48.44 ± 15.86		

### 3.4 Multivariable regression analysis

Multivariable linear regression analysis identified several factors independently associated with decision self-efficacy (Table 3). Education level showed a strong positive association with decision self-efficacy. Compared with participants with primary school education or below, those with junior high school education (β = 12.15, 95% CI: 9.84–14.45, *P* < 0.001) and high school education or above (β = 21.92, 95% CI: 17.05–26.79, *P* < 0.001) had significantly higher scores.

**Table 3:** Multiple regression analysis for factors associated with decision self-efficacy in Chinese adults with pneumoconiosis.

Characteristics	Options	β (95% CI)	*P*
Gender	Male	Reference	-
	Female	−1.83 (−6.33, 2.68)	0.425
Residence	Urban	Reference	-
	Town	0.79 (−1.97, 3.54)	0.575
	Rural	−1.96 (−4.83, 0.91)	0.180
Marital status	Married	Reference	-
	Other Widowed/Divorced	−18.57 (−22.27, −14.87)	< 0.001
Education	Primary school and below	Reference	-
	Junior high school	12.15 (9.84, 14.45)	< 0.001
	High school or above	21.92 (17.05, 26.79)	< 0.001
Healthcare payment	Employee Medical Insurance	Reference	-
	Out-of-pocket	3.11 (−2.77, 8.99)	0.298
	Resident Medical Insurance	−2.60 (−6.73, 1.52)	0.215
Monthly income	< 3000 yuan	Reference	-
	3000–5000 yuan	3.43 (0.67, 6.20)	0.015
	5000–10000 yuan	10.98 (7.41, 14.54)	< 0.001
	>10000 yuan	15.16 (9.37, 20.96)	< 0.001
Disease course	0–5 years	Reference	-
	6–10 years	2.23 (0.05, 4.40)	0.045
	>10 years	0.72 (−2.07, 3.51)	0.612
Stage	Stage I	Reference	-
	Stage II	−8.88 (−11.53, −6.24)	< 0.001
	Stage III	−19.56 (−23.13, −15.99)	< 0.001
Comorbidity	No	Reference	-
	Yes	−0.28 (−2.44, 1.88)	0.800
Rehabilitation exercise	Never	Reference	-
	Occasionally	−0.07 (−2.31, 2.17)	0.950
	Regularly	2.01 (−0.71, 4.72)	0.147
Home oxygen therapy	No	Reference	-
	Yes	−4.56 (−8.03, −1.08)	0.010
Anxiety (SAS)	No	Reference	-
	Yes	−4.26 (−7.09, −1.42)	0.003
Depression (SDS)	No	Reference	-
	Yes	−1.90 (−4.64, 0.84)	0.173

Monthly income was also positively associated with decision self-efficacy. Compared with participants earning less than 3,000 yuan per month, those earning 3,000–5,000 yuan (β = 3.43, 95% CI: 0.67–6.20, *P* = 0.015), 5,000–10,000 yuan (β = 10.98, 95% CI: 7.41–14.54, *P* < 0.001), and more than 10,000 yuan (β = 15.16, 95% CI: 9.37–20.96, *P* < 0.001) had progressively higher decision self-efficacy scores.

Disease severity was inversely associated with decision self-efficacy. Compared with stage I pneumoconiosis, stage II (β = −8.88, 95% CI: −11.53 to −6.24, *P* < 0.001) and stage III disease (β = −19.56, 95% CI: −23.13 to −15.99, *P* < 0.001) were associated with significantly lower scores.

Marital status was another significant factor, with widowed or divorced participants showing substantially lower decision self-efficacy than married participants (β = −18.57, 95% CI: −22.27 to −14.87, *P* < 0.001). In addition, home oxygen therapy (β = −4.56, 95% CI: −8.03 to −1.08, *P* = 0.010) and anxiety (β = −4.26, 95% CI: −7.09 to −1.42, *P* = 0.003) were independently associated with lower decision self-efficacy.

Gender, residence, healthcare payment type, comorbidity, rehabilitation exercise, disease duration, and depression were not independently associated with decision self-efficacy after adjustment.

### 3.5 Interaction between education level and monthly income

**Figure 2:**
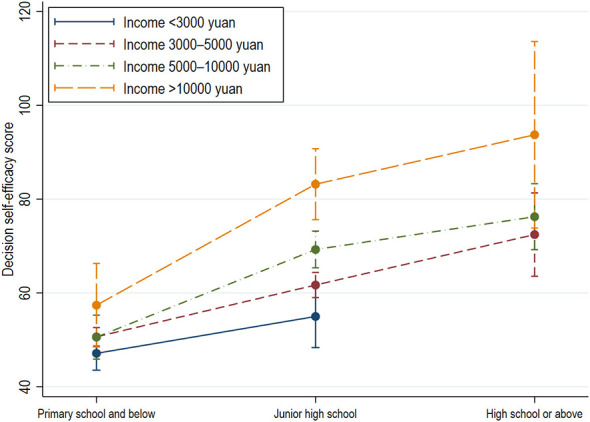
Interaction between education level and monthly income on decision self-efficacy score. Adjusted for education, monthly income, marital status, stage, home oxygen therapy and anxiety.

A significant interaction between education level and monthly income was observed (*P* for interaction = 0.033) (Figure 2, Supplementary Table 3). Specifically, participants with junior high school education and income >10,000 yuan had significantly higher decision self-efficacy (β = 17.10, 95% CI: 3.87–30.33). In contrast, interaction terms involving high school education or above were not significant, which may be attributed to insufficient sample size in the higher income groups.

## 4 Discussion

This study provides the first systematic assessment of decision-making self-efficacy in Chinese adults with pneumoconiosis. The mean self-efficacy score was 56.75 ± 17.54, reflecting a moderate-to-low level. Multivariable analysis identified higher education and income as independent positive predictors, while advanced disease stage, being widowed or divorced, home oxygen therapy, and anxiety were associated with lower self-efficacy. Notably, a significant interaction between education and income suggested that higher income amplified the positive effect of education on decision-making confidence.

The observed self-efficacy level among patients with pneumoconiosis is lower than that reported in previous studies among patients with cancer and individuals with multiple chronic conditions, as described by Berivan Yildiz et al. and Michele Peters et al., respectively (11, 12). The relatively low level of decision-making self-efficacy observed in patients with pneumoconiosis may be attributable to the long-standing under-recognition of this disease, limited research attention, and substantially lower public and clinical awareness compared with other chronic respiratory diseases, such as chronic obstructive pulmonary disease or bronchial asthma (13). These findings suggest that decision-making self-efficacy among patients with pneumoconiosis remains suboptimal and warrants further improvement. Nursing professionals and healthcare providers should therefore implement self-management interventions guided by self-efficacy theory and strengthen patient education and support to enhance patients' confidence and capacity to participate effectively in healthcare decision-making.

Our results further demonstrated that educational attainment, income level, disease stage, marital status, home oxygen therapy, and anxiety status were significant determinants of decision-making self-efficacy among patients with pneumoconiosis. Patients with higher educational levels, higher income, and those who were married generally exhibited greater decision-making self-efficacy. These findings are consistent with previous studies (9), which have shown that individuals with higher education and income tend to possess better health literacy, stronger information-seeking abilities, and greater access to healthcare resources. Such advantages facilitate clearer understanding of disease conditions and treatment options, reduce the cognitive burden associated with complex decisions, alleviate financial constraints, and enhance confidence and engagement in decision-making. In contrast, patients with lower educational attainment may struggle to comprehend medical information, while those with lower income often experience increased financial stress, which may exacerbate decisional anxiety and reduce willingness to participate actively in healthcare decisions. Accordingly, healthcare providers should adopt stratified, individualized, and multi-channel communication strategies tailored to patients' educational and economic backgrounds, ensuring that disease- and treatment-related information is presented in an accessible and comprehensible manner. Such approaches may improve health literacy and positive illness perceptions while minimizing information overload and financial stress, thereby facilitating higher-quality decision-making processes.

Notably, our study identified a significant positive dose–response relationship between income level and decision-making self-efficacy, with higher income associated with progressively higher self-efficacy scores. Previous research has indicated that patients experiencing financial hardship often face limited treatment options and restricted access to information, making them more prone to decisional hesitation and regret (14). Conversely, higher income is often associated with better social security coverage and greater capacity to invest in health-promoting resources or supportive interventions that facilitate disease management and recovery (5). Regarding marital status, married patients typically benefit from spousal or family companionship and emotional support, which can assist with information acquisition, participation in medical discussions, and sharing caregiving responsibilities. This form of family-based emotional and instrumental support may enhance patients' confidence in engaging in healthcare decisions (7). A recent systematic review by Wang et al. (15) highlighted that socioeconomic disadvantage (e.g., low education and low income) and lack of spousal or family support exacerbate psychological distress among patients with pneumoconiosis (15). Similarly, studies in patients with type 2 diabetes have shown that married individuals are more likely to engage family members in disease management and decision-making, receive greater social support, and demonstrate higher decision-making self-efficacy (16). These findings suggest that healthcare providers should pay particular attention to patients' social support needs during the decision-making process and offer additional resources, such as family-inclusive consultations and social work involvement, to encourage active participation in treatment decisions.

In addition, disease stage, home oxygen therapy, and anxiety status were also identified as important factors influencing decision-making self-efficacy. This is consistent with previous evidence indicating that patients with more severe chronic respiratory diseases tend to exhibit lower self-efficacy (17). Home oxygen therapy often reflects advanced disease severity and greater dependence on medical equipment, which may restrict mobility, increase social isolation, and negatively affect emotional wellbeing, thereby diminishing motivation to engage in decision-making. Prior research has shown that patients with chronic respiratory failure receiving long-term home oxygen therapy are particularly susceptible to anxiety (18). The detrimental impact of anxiety on decision-making has been well documented, with anxious patients more likely to experience heightened decisional conflict and reduced willingness to participate in healthcare decisions (19, 20).

Importantly, we observed a significant interaction between educational attainment and income level, suggesting that jointly considering patients' educational and economic circumstances may facilitate the identification of subgroups at heightened risk of low decision-making self-efficacy. Individuals with both low education and low income often face compounded barriers to accessing healthcare resources and information, further undermining their confidence in participating in decision-making (21, 22). Marginal effect analyses indicated that the combined effect of education and income was most pronounced among participants with moderate educational levels, implying that higher income may amplify the beneficial impact of education on decision confidence within this group. Owing to sample limitations, this interaction effect appeared less evident among those with higher education. Overall, our findings indicate that decision-making self-efficacy among patients with pneumoconiosis remains suboptimal, particularly among individuals with lower education, lower income, more advanced disease, lack of marital support, and elevated anxiety, warranting heightened attention from healthcare professionals.

Several limitations of this study should be acknowledged. First, the cross-sectional design precludes causal inference regarding the relationships between identified factors and decision-making self-efficacy. Second, participants were recruited from a single regional setting, which may limit the generalizability of the findings. Third, decision-making self-efficacy was assessed using self-reported questionnaires, introducing the potential for subjective bias. In addition, variables such as health literacy and social support were not measured, although they may play important roles in shaping self-efficacy. Future studies should adopt multicenter, prospective cohort or interventional designs with larger sample sizes and incorporate objective behavioral indicators to elucidate causal mechanisms. Based on the identified determinants, future research may also focus on developing and validating decision-support interventions tailored to patients with pneumoconiosis, including digital decision aids, multimedia health education programs, enhanced social support mechanisms, and interdisciplinary care models, with the goal of improving decision-making self-efficacy and patient-centered care. Furthermore, these findings may provide valuable guidance for healthcare providers and policymakers in designing patient-centered educational programs, communication strategies, and supportive healthcare systems aimed at enhancing treatment participation and the quality of healthcare decisions among patients with pneumoconiosis. By addressing educational, economic, and psychosocial barriers, targeted strategies can help optimize patient engagement and inform policies to support vulnerable patient populations.

## 5 Conclusion

In summary, decision-making self-efficacy among patients with pneumoconiosis is at a moderate-to-low level, and associated with educational levels, monthly income, marital status, disease severity, use of home oxygen therapy, and anxiety status. Enhancing patients' confidence and capacity to participate in healthcare decisions through targeted, tailored interventions is crucial to promoting shared decision-making, patient engagement, and improved health outcomes in this population. Future research should explore the effectiveness of targeted interventions in multicenter and longitudinal settings.

## Data Availability

The raw data supporting the conclusions of this article will be made available by the authors, without undue reservation.
