# Supplementary material for: Decision self-efficacy and its determinants among Chinese adults with pneumoconiosis: a cross-sectional study
Source: Front Public Health. 2026 Jun 30;14:1843585. doi: 10.3389/fpubh.2026.1843585 (PMC13364836; doi:10.3389/fpubh.2026.1843585)
Supplement: Supplementary file 1 [file Table_1.docx]

**Supplementary Table 1.** The items of decision self-efficacy.

| No | Description of the item | Value |
| --- | --- | --- |
| 1 | Get accurate information about my possible medical options. | Strongly Disagree: 0 points  Disagree: 1 point  Neutral: 2 points  Agree: 3 points  Strongly Agree: 4 points |
| 2 | Get information about the benefits of each option. |  |
| 3 | Get information about the risks and side effects of each option. |  |
| 4 | Be fully informed and able to make a choice. |  |
| 5 | Ask questions without feeling hesitant. |  |
| 6 | Express my concerns about each treatment option. |  |
| 7 | Actively seek support. |  |
| 8 | Understand which treatment option is best for me. |  |
| 9 | Handle unnecessary pressure from others when making a choice. |  |
| 10 | Let the healthcare team know what is best for me. |  |
| 11 | Delay the decision if I feel I need more time. |  |

**Supplementary table 2.** Distribution of Decision Self-Efficacy Scores.

| No | Description of the item | Mean±SD* |
| --- | --- | --- |
| 1 | Get accurate information about my possible medical options. | 2.32±0.90 |
| 2 | Get information about the benefits of each option. | 2.29±0.88 |
| 3 | Get information about the risks and side effects of each option. | 2.21±0.89 |
| 4 | Be fully informed and able to make a choice. | 2.34±0.94 |
| 5 | Ask questions without feeling hesitant. | 2.19±0.94 |
| 6 | Express my concerns about each treatment option. | 2.25±0.90 |
| 7 | Actively seek support. | 2.35±0.88 |
| 8 | Understand which treatment option is best for me. | 2.26±0.97 |
| 9 | Handle unnecessary pressure from others when making a choice. | 2.24±0.94 |
| 10 | Let the healthcare team know what is best for me. | 2.26±0.92 |
| 11 | Delay the decision if I feel I need more time. | 2.28±0.91 |
|  | Total Score | 56.75±17.54 |

* According to the Shapiro-Francia test, both the total score and all individual items are normally distributed (p > 0.05).

**Supplementary table 3.** Interaction between education level and monthly income on decision self-efficacy score

| Variables | β (95% CI) | P |
| --- | --- | --- |
| Education |  |  |
| Primary school and below | Reference |  |
| Junior high school | 9.30,(2.10, 16.49) | 0.012 |
| High school or above | 33.99,(13.33, 54.66) | 0.001 |
| Monthly Income |  |  |
| <3000 yuan | Reference |  |
| 3000-5000 yuan | 3.78,(-0.11, 7.66) | 0.057 |
| 5000-10000 yuan | 4.35,(-1.24, 9.95) | 0.127 |
| >10000 yuan | 10.14,(0.98, 19.31) | 0.030 |
| Education*Monthly Income |  |  |
| (Junior high school)*(3000-5000 yuan) | 1.34,(-6.51, 9.19) | 0.736 |
| (Junior high school)*(5000-10000 yuan) | 9.02,(-0.25, 18.30) | 0.056 |
| (Junior high school)*(>10000 yuan) | 17.10,(3.87, 30.33) | 0.012 |
| (High school or above)*(3000-5000 yuan) | -11.01,(-33.46, 11.44) | 0.335 |
| (High school or above)*(5000-10000 yuan) | -9.41,(-31.64, 12.82) | 0.405 |
| (High school or above)*(>10000 yuan) | / | / |

* Adjusted for marital status, stage, home oxygen therapy and anxiety.
